# A retrospective observational study of vitamin D levels in patients within the Tameside and Glossop early intervention in psychosis team

**DOI:** 10.1192/bjo.2021.701

**Published:** 2021-06-18

**Authors:** Emily Kaye, Pete Parker, Thomas Fyall, Katie Arrowsmith, Holly Hark, Samei Ahmed Huda

**Affiliations:** Tameside and Glossop Early Intervention Team

## Abstract

**Aims:**

Growing evidence indicates that Vitamin D deficiency is associated with psychotic symptoms. Although evidence suggesting a causal relationship is limited, theories regarding neuro-inflammatory modulation are promising. Alternatively, deficiency may signify chronic illness or poor functioning. Nevertheless, Vitamin D levels below 50nmol/L increase the risk of osteoporosis, muscle weakness, falls and fractures, thus identification and treatment are important.

The association between Vitamin D levels in patients within the Tameside Early Intervention in Psychosis Team (EIT) was studied, hypothesising a strong correlation.

**Method:**

The records of all patients in the EIT as of 01/07/2020, over the age of 16 years old (n = 183), were studied. The first Vitamin D level taken while under the EIT and the CGI scores closest to the date of this level were recorded. Vitamin D levels of 25nmol/L and under were classified as deficient, levels of 25.1 - 50nmol/L were insufficient.

**Result:**

45.90% (n = 84) of patients did not have their levels recorded. Of the 55% (n = 99) patients who had Vitamin D levels recorded, 49.50% (n = 49) were insufficient and 22.22% (n = 22) were deficient. Therefore, only 28.28% (n = 28) had either optimal or sufficient Vitamin D levels. The majority of Vitamin D levels were taken in Autumn (36.46% n = 36).

75.76% (n = 75) of patients had both vitamin D levels and CGI scores recorded, with an average of 35.65 days between date level and score recorded. A weak negative correlation between overall CGI scores and vitamin D level was calculated, producing Spearman R Correlation Coefficient of -0.15.

**Conclusion:**

Almost 3/4 of the studied patients being assessed for psychotic symptoms had either insufficient or deficient levels of Vitamin D. The correlation between symptom severity and Vitamin D level was weak however. While we cannot comment on the causality of the relationship, it appears that there is an association between our studied patient group and Vitamin D insufficiency.

The evidence to suggest that supplementation can reduce psychotic symptoms is limited however, supplementation can reduce the risk of osteoporosis and falls, therefore would improve patient care. Only 55% of the patients within the EIT had their Vitamin D levels tested. As a result of this study, the authors recommend that all patients in the EIT have their Vitamin D levels tested as part of their psychosis assessment.

The study is limited due to low numbers of patients studied and the fact that recorded CGI scores were often recorded at a later date to Vitamin D levels.

